# The Effect of Negative Pressure Wound Therapy with and without Instillation on Mature Biofilms In Vitro

**DOI:** 10.3390/ma11050811

**Published:** 2018-05-16

**Authors:** Shamaila Tahir, Matthew Malone, Honghua Hu, Anand Deva, Karen Vickery

**Affiliations:** 1 Surgical Infection Research Group, Faculty of Medicine and Health Sciences, Macquarie University, Sydney 2109, Australia; helen.hu@mq.edu.au (H.H.); anand.deva@mq.edu.au (A.D.); karen.vickery@mq.edu.au (K.V.); 2 Infectious Diseases and Microbiology, School of Medicine, Western Sydney University, Sydney 2751, Australia; matthew.malone@westernsydney.edu.au; 3 Liverpool Diabetes Collaborative Research Unit, Ingham Institute of Applied Medical Research, Sydney 2170, Australia; 4 High Risk Foot Service, Liverpool Hospital, South West Sydney LHD, Sydney 2170, Australia

**Keywords:** biofilm, chronic wounds, instillation therapy, in vitro

## Abstract

Background: To investigate the effect of negative pressure wound therapy (NPWT) with and without instillation (NPWTi) on in vitro mature biofilm. Methods: Mature biofilms of *Pseudomonas aeruginosa* and *Staphylococcus aureus* were grown under shear (130 rpm) on polycarbonate coupons in a CDC biofilm reactor for 3 days. Coupons containing biofilms were placed in a sterile petri dish and sealed using NPWT or NPWTi. Coupons were exposed to treatment for 24 h with NPWT alone or with instillation of: Povidone iodine solution (PVP-I) (10% *w*/*v* equivalent to 1% *w*/*v* available iodine, BETADINE^®^, Mundipharma, Singapore), surfactant based antimicrobial solution with polyhexamethylene biguanide (SBPHMB) (Prontosan^®^, B Braun Medical, Melsungen, Germany), Gentamicin 1 µg/mL (GM) (G1264 Sigma-Aldrich Pty Ltd., Castle Hill, Australia) Rifampicin 24 µg/mL (RF) (R3501 Sigma-Aldrich Pty Ltd., Castle Hill, Australia) and NaCl 0.9% (Baxter, Deerfield, IL, USA). Bacterial cell viability and biofilm architecture pre-and post-treatment were assessed using colony forming units (cfu), Live/Dead viability staining, confocal laser scanning microscopy (CLSM) and scanning electron microscopy (SEM). Results: Significant reductions were obtained in *S. aureus* biofilm thickness (65%) and mass (47%) when treated with NPWTi as compared to NPWT only. NPWTi with instillation of SBPHMB, PVP-I and RF achieved between 2 and 8 log_10_ reductions against *S. aureus* biofilm (*p* < 0.05–0.001). Conversely, PVP-I and SBMO achieved a 3.5 log_10_ reduction against *P. aeruginosa* (*p* < 0.05). Conclusions: NPWT alters biofilm architecture by reducing biofilm thickness and mass, but this does not affect bacterial cell viability. NPWT with instillation of certain antimicrobials solutions may provide a further synergistic effect in reducing the number of viable biofilm microorganisms. Our in vitro model may be used for screening the effectiveness of antimicrobials used under instillation prior to animal or human studies.

## 1. Introduction

The causality of a wound that experiences a delay in healing can be multifactorial and attributed to factors such as local tissue hypoxia/poor perfusion, repetitive ischemia-reperfusion injury [1], microbial infection [2], inadequate offloading or compression therapy [3]. Perhaps the most significant of these factors are the cases where chronic wounds become complicated by pathogenic microorganisms. These may exist as planktonic rapidly dividing cells that invade host tissues and induce an acute infection [4]. Conversely, some microorganisms that complicate chronic wounds may alter their phenotype, differing markedly in both their physiology and activity. These microorganisms are sessile, attach to surfaces or other microorganisms, form aggregates, and regulate the production of an extracellular polymeric substance (biofilm) [5]. The hallmark features of these microorganisms are their tolerance to antimicrobials, the host immune responses and environmental stresses. 

These wounds are a challenge for any clinician and ensuring their resolution can often involve a complex array of pathways that may involve surgical or sharp conservative debridement of any infected non-viable tissue. Even in this scenario the ability for a surgeon/clinician to remove all non-viable tissue and any microorganisms not visible to the naked eye is likely not possible [6]. Post-surgical debridement wound care is therefore a critical step to ensure a newly ‘acute’ wound continues through the orderly continuum of repair. To augment this process negative pressure wound therapy with instillation (NPWTi) and dwell time is an adjunctive treatment modality for selected complex wounds complicated by invasive infection or extensive biofilm [7,8].

Evidence for NPWT with or without instillation/dwell time on the microbial load of wounds is limited [9,10,11] with little data available for its action/s against microbial biofilms [12]. Previously our group demonstrated that NPWT resulted in a physical disruption to biofilm architecture [13]. This change resulted in a synergism between NPWT and a solid dressing (silver impregnated foam) eradicating an in vitro biofilm [14]. In this study we aim to test the effectiveness of NPWT with instillation and dwell time of topical antimicrobial solutions, against 3-day mature *S. aureus* and *P. aeruginosa* biofilms. We hypothesize that NPWT alters biofilm architecture and thus improves penetration of antimicrobials through the extracellular polymeric substance of biofilm forming microorganisms.

## 2. Materials and Methods

### 2.1. Bacterial Test Strains

Biofilm forming reference strains utilized in vitro were *S. aureus* (ATCC^®^ 25923™), (methicillin-sensitive *S. aureus* (MSSA) and *P. aeruginosa* (ATCC^®^ 25619™).

### 2.2. Solutions Used for Instillation Therapy

Details regarding the solutions used, any incorporated antimicrobials and tested concentration levels, and their respective manufacturers are noted in the Supplementary Materials (Table S1). Briefly, surfactant based antimicrobial solution with polyhexamethylene biguanide (SBPHMB; Prontosan^®^, B Braun Medical, Melsungen, Germany), povidone iodine (PVP-I) antimicrobial solution 10% *w*/*v* equivalent to 1% *w*/*v* available iodine (BETADINE^®^, Mundipharma, Singapore), Saline (NaCl) 0.9% (Baxter, Deerfield, IL, USA). The systemic antimicrobials tested were, gentamicin (GM) 1 µg/mL and Rifampicin (RF) 24 µg/mL, both diluted in NaCl 0.9% (Baxter International, Deerfield, IL, USA).

### 2.3. In Vitro CDC Biofilm Reactor

*P. aeruginosa* and *S. aureus* were grown separately under shear (130 rpm) at 35 °C on 24 removable polycarbonate coupons in a CDC biofilm reactor (BioSurface Technologies Corp., Bozeman, MT, USA). *S. aureus* biofilm was grown in 15 g/L (50%) tryptone soya broth (TSB) (Sigma Aldrich, St. Louis, MO, USA) in batch phase for 24 h and then replaced with fresh media 6 g/L (20% TSB) flowing through the chamber at 80 mL/h for a further 48 h. *P. aeruginosa* was grown in 600 mg/L (2%) TSB in batch phase for 24 h and then with fresh media (TSB 2%) flowing through the chamber at 80 mL/h for a further 48 h. Coupons were harvested by washing gently, three times, in phosphate buffered saline (PBS) to remove loosely attached and planktonic bacteria. The number of bacteria per coupon was 3.52 × 10^7^ and 2.3 × 10^7^ for *S. aureus* and *P. aeruginosa,* respectively. Bacterial biofilm was gently scraped off from the outer side of each coupon using a 12.5% sodium hypochlorite-soaked paper towel, and then again washed three times in TSB to remove residual chlorine.

### 2.4. In Vitro NPWTi Model

The NPWTi utilized in this study was the V.A.C. Ulta negative pressure wound therapy system (Acelity, San Antonio, TX, USA) incorporating the V.A.C. Veraflo therapy that allows the controlled instillation of topical solutions. Modifications to the system were necessary due to the tubular shape of the V.A.C. Veraflo dressing system, in keeping with previously published reports [13]. 

**Figure 1:**
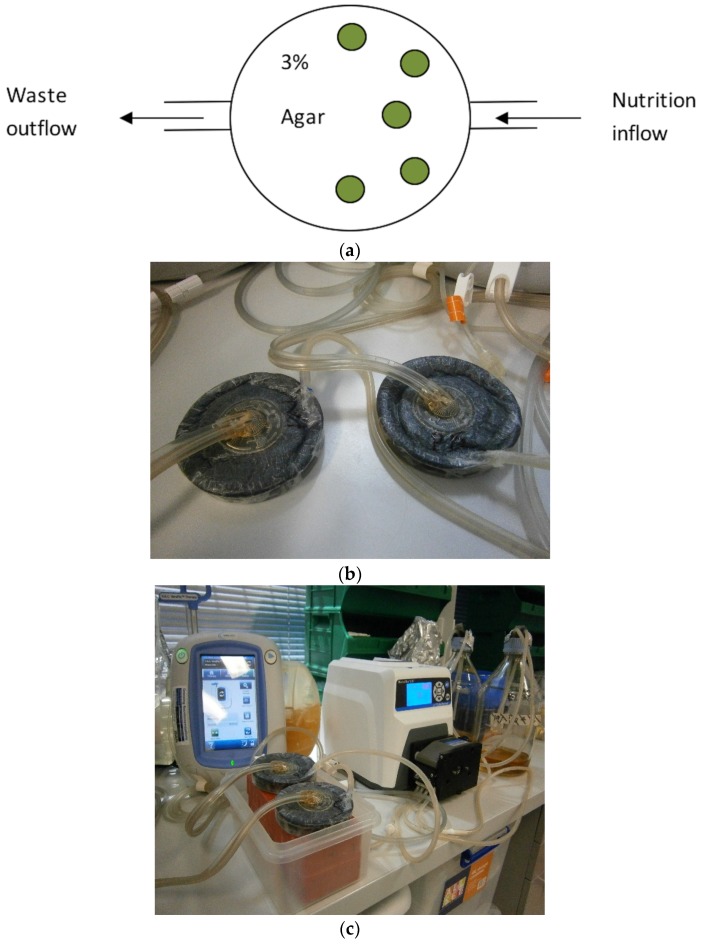
(**a**) Schematic presentation of modified wound model with polycarbonate coupons (green). (**b**) New wound model with Veraflo dressing system. (**c**) Experimental setup of instillation + V.A.C. therapy. Green circles represent biofilm coated polycarbonate coupons.

Five biofilm containing coupons were placed on top of 3% bacteriological agar (Thermo Scientific, Basingstoke, UK) in a sterile petri dish. The sterile NPWT dressing (V.A.C.^®^ GRANUFOAM™) were added on top of the coupons until the petri dish was completely full, thus ensuring equal pressure application to all five biofilm covered coupons. An airtight seal was produced using a sterile semi-impervious dressing (V.A.C.^®^ dressing system). In order to emulate wound exudate, coupons were bathed with TSB (30 g/L) at a flow rate of 40 mL/h via an inflow channel [15,16]. Excess fluid was drained via a gravity drainage tube, situated on the opposite side from the nutrition in-flow, for chambers not subjected to NPWT and via a centrally placed V.A.C.^®^ Veralink Cassette for chambers subjected to NPWT (Figure 1).

Five coupons for each test antimicrobial solution were exposed to the following treatment variables: (i) control with no treatment; (ii) NPWT alone with no instillation; (iii) instillation of antimicrobial plus continuous NPWT at 125 mmHg (except during the 20 min instillation treatment periods); and (iv) instillation of antimicrobial solution with no NPWT. The instillation cycles were as follows: instillation every 6 h with 35 mL of saline or test antimicrobial with a 20 min dwell time in a 24 h time period. Instillation with TSB was then continued for another 6 h before harvesting.

### 2.5. Bacterial Viability cfu/log_10_

At the end of each treatment period, the numbers of residual bacterial colony forming units (cfu) per coupon were tested in triplicate by sonication in an ultrasonic bath (Soniclean, Stepney, Australia) for 10 min with a sweeping frequency of 42–47 kH at 20 °C.

Coupons were then vortexed for two min in 2 mL of PBS followed by sequential 10-fold dilution and plate count. Pre- and post-exposure average cfu/coupon was expressed as log_10_. 

### 2.6. Confocal Laser Scanning Microscopy

Bacterial cell viability pre- and post-exposure was also assessed using *Bac*Light™ (Live/Dead Bacterial Viability Kit, 7012, Molecular Probes, Invitrogen, Carlsbad, CA, USA) in conjunction with confocal laser scanning microscopy (CLSM) (Olympus FluoView™ FV1000, Tokyo, Japan). Following staining, coupons were fixed with 4% paraformaldehyde for 1 h and washed thrice with PBS for 10 min. 2D images were obtained within 24–48 h of staining. 3D images were obtained from three separate areas per coupon. Images were built with 0.2 µm optical sections and analyzed for average thickness, biofilm mass and percentage of viable cells, using the IMARIS 7.7.2 software (Bitplane, Zurich, Switzerland) and ImageJ program (scriptable Java application for scientific image processing). A 63× water immersion objective lens was used to capture images with reduced background noise at 10×, 20× and 40× magnifications. To minimize image artefacts these dual labelled (Syto-9 and propidium iodide) samples were sequentially scanned at 488 nm fluorescence excitation (green emission) and then at 543 nm (red emission) collected in the green and red regions, respectively. Line averaging (×2) was used to capture images with reduced noise. 

Biofilm architecture was analyzed using IMARIS (Bitplane AG, Zurich, Switzerland) software to quantify 3-D CLSM images by: (1) Average thickness is the distance (μm) between the top of a biofilm and the substratum on which the biofilm resides. It provides a measure of the spatial size of biofilm; (2) Average biofilm biomass (µm^3^), is defined as the volume of bacterial cells below µm^2^ area. The value excludes the non-cellular components (e.g., EPS and water channels) of biofilm volume.

### 2.7. Scanning Electron Microscopy

For SEM, one coupon each from selected antimicrobial treatment were fixed in 3% glutaraldehyde, dehydrated through serial dilutions of ethanol and then immersed in hexamethyldisilazane (Polysciences Inc., Warrington, FL, USA) for 10 min before being aspirated dry and air dried for at least 48 h. Coupons were then mounted on specimen stubs, gold coated and examined at low and high magnifications (JOEL 6480LA SEM, Tokyo, Japan).

#### Statistical Analysis

Statistical analysis on cfu data was performed using the Sigma Plot 11 statistical program (Scientific Graphing Software: SigmaPlot^®^ Version 11 by Systat Software, Inc., San Jose, CA, USA). Pre and post bacterial viability between treatment groups were analyzed by performing one-way analysis of variance (ANOVA). For non-normally distributed data a Kruskal-Wallis one-way analysis of variance on ranks was performed, and if significant, the Tukey test for all pair wise multiple comparisons were conducted to determine which treatment groups were significantly different from each other. 

## 3. Results

All experiments were conducted over a 24 h test period. Control coupons of *S. aureus* and *P. aeruginosa* receiving no treatment increased in the number of biofilm bacteria from a starting cfu of 7.4 log_10_ cfu/coupon to 8.3 log_10_ cfu/coupon (0.9 log_10_ cfu/coupon increase, *p* = 1.0). The effects of NPWT, NPWTi and instillation alone on bacterial viability after 24 h are reported.

### 3.1. NPWT on Bacterial Viability cfu/log_10_

NPWT had little effect on *S. aureus* biofilms demonstrating a 1.2 log_10_ cfu/coupon reduction (control no treatment = 7.4 log_10_ cfu/coupon vs. NPWT = 6.2 log_10_ cfu/coupon *p* > 1.0) while numbers increased in the case of *P. aeruginosa* by 0.7 log_10_ cfu/coupon (control no treatment = 7.4 log_10_ cfu/coupon vs. NPWT = 8.1 log_10_ cfu/coupon *p* > 1.0). 

### 3.2. Instillation Alone vs. NPWTi on Bacterial Viability cfu/log_10_

**Figure 2:**
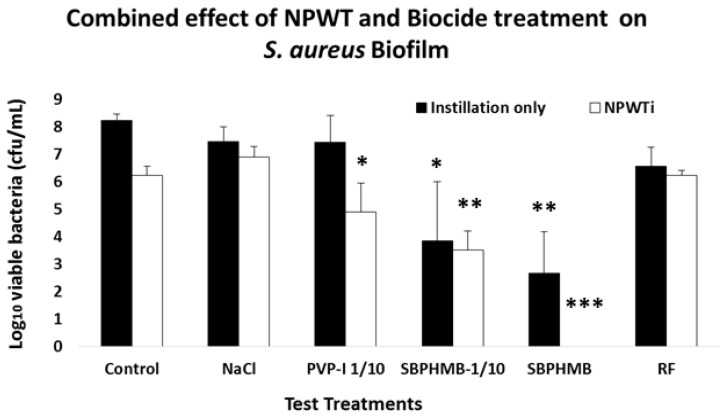
Mean log_10_ reduction of colony forming units (cfu) of *S. aureus* remaining on coupons following treatment with instillation alone or negative pressure wound therapy with instillation (NPWTi). Statistically significant from controls is shown by *, *p* value < 0.001 = (***), *p* value < 0.01 = (**), *p* value < 0.05 = (*).

**Figure 3:**
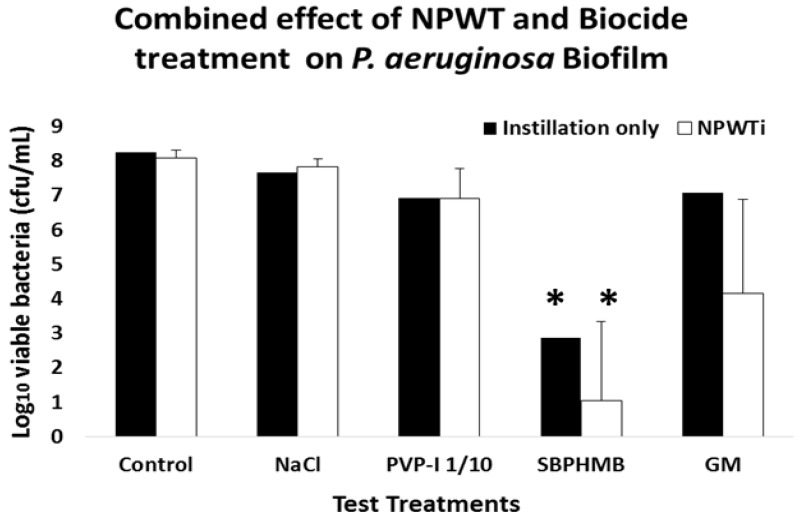
Mean log_10_ reduction of cfu of *P. aeruginosa* remaining on coupons following treatment with (*n* = 5) and without (*n* = 5) application of topical negative pressure. Statistically significant from controls is shown by *p* value < 0.01 = (**)*.*

Bacterial viability of *S. aureus* and *P. aeruginosa* following Instillation or NPWTi are reported in Figure 2 and Figure 3 and Table S2. Instillation with NaCl or PVP-I 1/10 demonstrated an equal reduction in *S. aureus* biofilms (0.8 log_10_ cfu/coupon, *p* = 0.2). When challenged against *P. aeruginosa* biofilms, NaCl reduced cfu by 0.6 log_10_ per coupon and PVP-I reduced cfu by 1.6 log_10_ per coupon (*p* = 0.1). SBPHMB was highly effective in reducing both *S. aureus* (5.6 log_10_ cfu/coupon *p* < 0.001) and *P. aeruginosa* biofilms (5.4 log_10_ cfu/coupon *p* = 0.01). RF achieved a 1.7 log_10_ cfu/coupon reduction against *S. aureus* (*p* = 0.09) and GM achieved a 1.22 log reduction cfu/coupon against *P. aeruginosa* (*p* = 0.1).

NPWTi demonstrated significant increases in effectiveness against both *S. aureus* and *P. aeruginosa* when compared to instillation alone. When challenged against *S. aureus*, NPWTi using NaCl demonstrated a 1.4 log_10_ cfu/coupon reduction, (*p* = 0.1), PVP-I a 3.4 log_10_ cfu/coupon reduction (*p* = 0.05), SBPHMB an 8.3 log_10_ cfu/coupon reduction (*p* = 0.001) and RF a 2.1 log_10_ cfu/coupon reduction (*p* = 0.05). For *P. aeruginosa*, NPWTi using NaCl demonstrated a 0.4 log_10_ cfu/coupon (*p* = 0.1), PVP-I a 1.3 log_10_ cfu/coupon reduction (*p* = 0.2), SBPHMB a 7.3 log_10_ cfu/coupon reduction (*p* < 0.0001) and GM a 4.1 log_10_ cfu/coupon reduction (*p* = 0.01).

### 3.3. Microscopy

**Figure 4:**
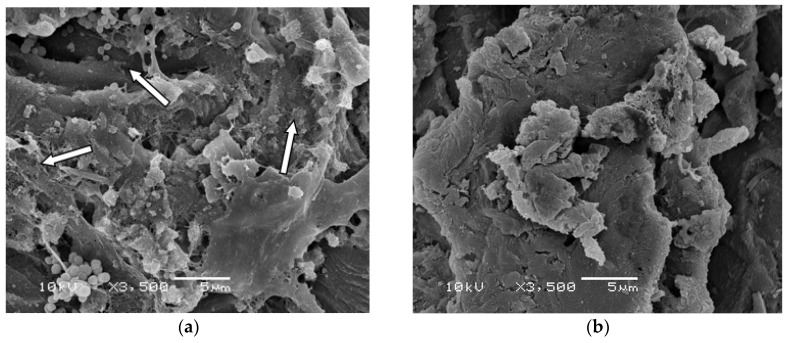

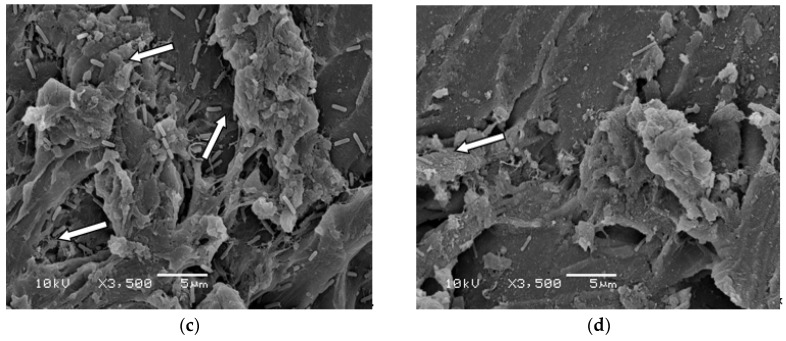
(**a**) SEM images of *S. aureus* biofilm coupon using SBPHMB/Instillation only for 24-h identifies dense coccoid bacteria embedded in EPS after treatment; (**b**) demonstrates an overall reduction in *S. aureus* biofilm after 24-h treatment with NPWTi using SBPHMB; (**c**) illustrates *P. aeruginosa* biofilm coupon using SPHMB/Instillation only for 24-h; (**d**) after 24-h treatment with NPWTi using SBPHMB. Arrows indicate biofilm aggregates.

Given that SBPHMB demonstrated a greater efficacy than all other antimicrobials used in our biofilm models, we explored this agent in greater detail using both SEM and confocal microscopy with LIVE/DEAD stain. SEM images of *S. aureus* and *P. aeruginosa* biofilms from coupons undergoing instillation only with SBPHMB or NPWTi using SBPHMB are depicted in Figure 4. Minimal changes in biofilm structure of *S. aureus* and *P. aeruginosa* are noted in the SEM images (Figure 4a,c) following instillation of SBPHMB for 24 h. SEM identified dense coccoid and rod shaped microbial aggregates, respectively, embedded in a thick continuous EPS. Therefore, post-treatment with instillation only was ineffective. In contrast, SEM images of NPWTi using SBPHMB treated coupons showed significant reductions in biofilm EPS and in the number of microbial aggregates (Figure 4b,d). This was particularly evident for *S. aureus* biofilms, which demonstrated complete eradication of cocci aggregates and extracellular polymeric substance (EPS) (Figure 4b).

**Figure 5:**
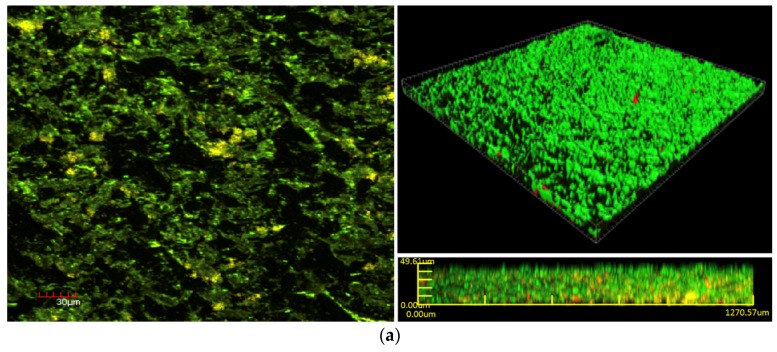

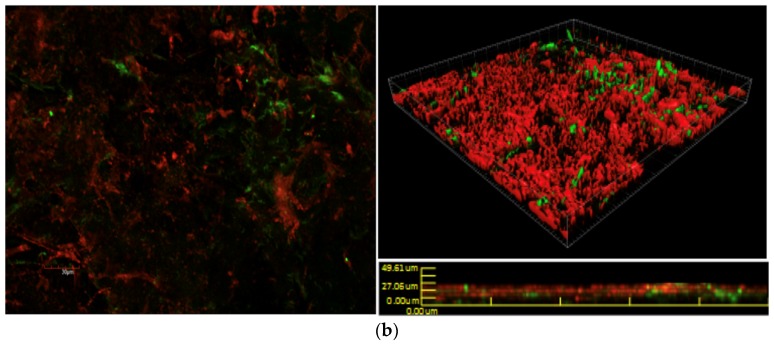
CLSM (30 μm) images of *S. aureus* biofilm with LIVE/DEAD^®^ BacLight™ Bacterial Viability Kit, (**a**) pre-treatment with NPWTi using SBPHMB and (**b**) post-treatment with NPWTi using SBPHMB. Live bacteria are stained green and dead bacteria are stained red.

**Figure 6:**
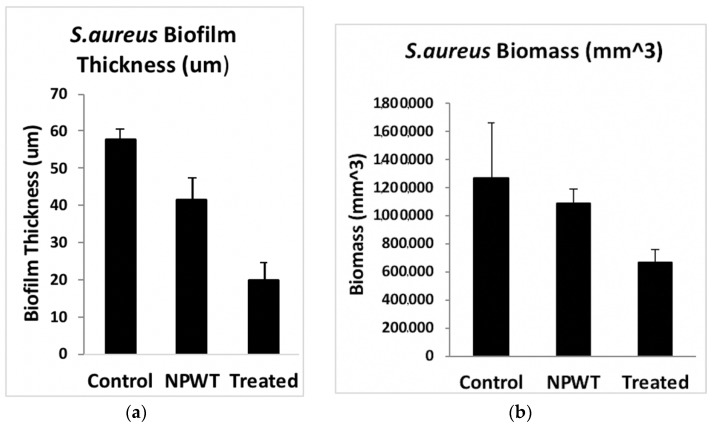
Demonstrates >60% reduction in *S. aureus* biofilm thickness (**a**) and >45% reduction in *S. aureus* biofilm biomass (**b**). *S. aureus* biofilm biomass pre-treatment NPWTi using SBPHMB.

**Figure 7:**
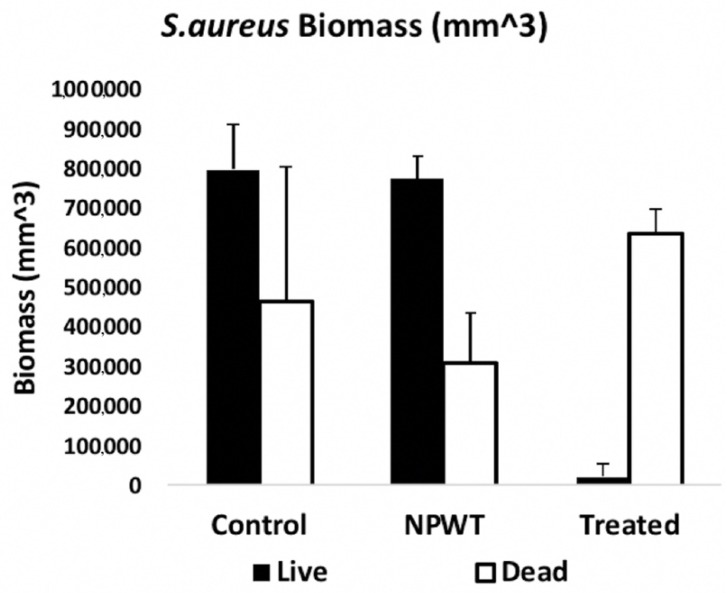
Demonstrates a 97% reduction in *S. aureus* biomass of live/dead cells after NPWTi using SBPHMB.

Bacterial cell viability pre- and post-exposure of NPWTi with SBPHMB were analyzed using LIVE/DEAD stain with CLSM. This identified up to 97% reduction in live cells while live cells reduced by 3% in NPWT only treated *S. aureus* biofilm (Figure 5). The effects of NPWT with or without instillation on biofilm thickness and biomass are noted in Figure 6 and Figure 7. Control coupons receiving no treatment had an average biofilm thickness of 57 μm and an average biomass of 1,264,111 µm^3^. Coupons with *S. aureus* biofilms treated with NPWT alone had average thickness of 41 μm and biomass of 1,081,458 µm^3^. In comparison, coupons with *S. aureus* biofilms treated with NPWTi SBPHMB experienced a 65% reduction in biofilm thickness (pre-treatment biofilm thickness = 57 μm versus post-treatment biofilm thickness = 19.8 μm, *p* < 0.0003), and a 48% reduction in total biofilm biomass (pre-treatment biofilm biomass = 1,264,111 µm^3^ versus post-treatment biofilm biomass = 770,968 µm^3^, *p* = 0.05). 

## 4. Discussion

NPWTi is reported to improve wound healing over NPWT alone, by enhancing autolytic and mechanical debridement and reducing the microbial load [17]. However there has been limited information detailing the increased efficacy of NPWTi over standard negative pressure with respect to microbial biofilms. The outcomes of this study confirm our previous work [13] and clearly demonstrate that NPWT alters in vitro biofilm architecture, reduces biofilm thickness and biomass, and decreases the diffusion distances for the penetration of antimicrobials through biofilms. We suggest that this action likely creates a synergy with antimicrobial solutions used under NPWTi (some antimicrobials have higher efficacy than others). 

Given there has been a tremendous surge in research exploring anti-biofilm strategies for use in healthcare associated chronic infections, various agents have been explored that have included peptides, antiseptics, oral and topical antimicrobials. The methods of delivering these treatments have also varied and have included coatings, drug eluting, wound gels, nanoparticles, irrigations, and solutions. To complicate the picture, various methodologies to quantify outcomes measures have been used both in vitro and in animal models, and this lack of standardization makes comparing the results of different studies difficult [18]. The in vitro model utilized in this study is standardized, reproducible and easy-to-use. Furthermore this in vitro model offers a useful screening tool to identify potential antimicrobial solutions with greater efficacy against microbial biofilms when used under NPWTi, prior to undertaking animal or human studies.

These studies are often more complex requiring both a skilled laboratory/research group in addition to being costly. This has likely contributed to the limited studies to date (either in vitro or animal models), which have explored NPWTi using saline solutions or antimicrobial solutions against mature biofilms. Singh et al. [19] used an in vivo animal model to demonstrate the role of NPWT with antimicrobial instillation (Prontosan^®^, B Braun Medical, Melsungen, Germany) against clearance of infection and biofilm formation of infected spinal implants compared to traditional treatment modalities. A biofilm-forming methicillin-resistant *S. aureus* strain was grown for seven days in vivo, on implanted titanium rods that were then subjected to either wet to dry dressings (control arm) or NPWTi for a further seven days. The mean bacterial loads and presence of biofilm were lower in pigs receiving NPWTi (the experimental group was 6647 cfus/mL and 13,303 cfus/mL in the control group and SEM revealed the presence of uniform biofilm formation across the surface of control group instrumentation, the experimental group was positive for biofilm formation but with many skipped areas with no biofilm).

In another porcine skin explant model using NPWTi of various antimicrobial solutions, Phillips et al. [12] identified that SBPHMB and PVP-I reduced 3-day mature biofilms of *P. aeruginosa* by 4 log_10_ and 5 log_10_ respectively. In concluding, the authors hypothesize the potential synergy experienced was due to macrodistortion/microdistortion forces produced by negative pressure therapy, altering the biofilm EPS matrix structure sufficiently to enhance the penetration of the antimicrobial agents into the biofilm. 

This can be clearly demonstrated by our confocal microscopy and in previous work by our group [13], which illustrates changes to biofilm architecture under negative pressure. We illustrate that NPWT significantly reduced biofilm thickness but has no effect on biomass which is concordance with viability results showing no reduction in bacterial numbers. In other words, the NPWT physically disrupts biofilm architecture by compressing it but does not kill the bacteria. In comparison, NPWTi significantly reduced both *S. aureus* biofilm thickness (reduced by 65%) and total biofilm mass (reduced by 48%), which was mirrored by a significant reduction in cell viability. This suggests a potential synergism between NPWT and the antimicrobial solution. The study results reflect that the synergism between antimicrobials and NPWT seems dependent upon bacterial species in addition to the type of antimicrobial used. For example, at the concentrations used for Rifampicin during instillation only, there was little to no effect on *S. aureus* biofilms even when RF was used under NPWTi.

## 5. Conclusions

With regards to wound care products in general, the majority of data on anti-biofilm strategies have been undertaken in vitro. This represents a challenge for clinicians where in vitro data may be based on laboratory methods that lack both standardized approaches and clinical relevance. The results obtained from in vitro testing which identify an effective wound care product, may therefore not translate into the same efficacy or outcomes when used clinically in vivo. Our in vitro model allows the simple and effective screening of antimicrobial solutions that may be used by clinicians as part of NPWTi therapy. However, the in vitro data generated from this model are a necessary precursor to further testing in more clinically relevant scenarios (that are often significantly more expensive) such as animal models or human studies, where results can be correlated.

## Supplementary Materials

The following are available online at http://www.mdpi.com/1996-1944/11/5/811/s1, Table S1: Biocide clinical concentration and concentration used for in vitro wound model efficacy testing. Betadine and Prontosan concentration expressed as dilution of commercially available product. Table S2: Mean number of *S. aureus* and *P. aeruginosa* remaining on no treatment coupons and treatment coupons following four instillations of saline or biocide in 24 h with and without application of NPWT. Controls for *S. aureus* is 4.23 × 10^7^ and for *P. aeruginosa* is 2.3 × 10^7^. Number of samples, *n* = 5. Statistically significant from controls is shown by *****, *p* value < 0.001 = (*******), *p* value < 0.01 = (******), *p* value < 0.05 = (*****).
