# Supplementary material for: The Effect of Negative Pressure Wound Therapy with and without Instillation on Mature Biofilms In Vitro
Source: Materials (Basel). 2018 May 16;11(5):811. doi: 10.3390/ma11050811 (PMC5978188; doi:10.3390/ma11050811)
Supplement: Supplementary file 1 [file materials-11-00811-s001.pdf]

Supplementary Materials

The Effect of Negative Pressure Wound Therapy with and without Instillation on Mature Biofilms In Vitro

Shamaila Tahir [1,*], Matthew Malone [2,3,4], Honghua Hu [1], Anand Deva [1] and Karen Vickery [1]

[1] Surgical Infection Research Group, Faculty of Medicine and Health Sciences, Macquarie University, Sydney 2109, Australia; helen.hu@mq.edu.au (H.H.); anand.deva@mq.edu.au (A.D.); karen.vickery@mq.edu.au (K.V.)
[2] Infectious Diseases and Microbiology, School of Medicine, Western Sydney University, Sydney 2751, Australia; matthew.malone@westernsydney.edu.au
[3] Liverpool Diabetes Collaborative Research Unit, Ingham Institute of Applied Medical Research, Sydney 2170, Australia
[4] High Risk Foot Service, Liverpool Hospital, South West Sydney LHD, Sydney 2170, Australia
* Correspondence: shamaila.tahir@students.mq.edu.au

Table S1. Biocide clinical concentration and concentration used for *in vitro* wound model efficacy testing. Betadine and Prontosan concentration expressed as dilution of commercially available product.

Test Antimicrobials	Clinical Doses	Concentration Tested for *S. aureus* Biofilm	Concentration Tested for *P. aeruginosa* Biofilm
Betadine (Povidone-iodine 10%w/v) topical	1/5	1/10	1/10
Prontosan (0.1% Polyhexamethylene biguanide and 0.1% Betaine) topical wash	Neat	Neat and 1/10	Neat
Gentamicin solution	0.5–50 µg/mL	-	1 µg/mL
Rifampicin injectable (8.5 mg/kg)	120 µg/mL (blood conc.)	24 µg/ml	-

Table S2. Mean number of *S. aureus* and *P. aeruginosa* remaining on no treatment coupons and treatment coupons following four instillations of saline or biocide in 24 h with and without application of NPWT. Controls for *S. aureus* is 4.23×10^7 and for *P. aeruginosa* is 2.3×10^7. Number of samples, *n* = 5. Statistically significant from controls is shown by *, P value < 0.001 = (***), P value < 0.01 = (**), P value < 0.05 = (*).

Treatment	Mean Number of Colony Forming Units (CFU) of *S. aureus*		Mean Number of Colony Forming Units (CFU) of *P. aeruginosa*	
	No NPWT	NPWT	No NPWT	NPWT
Nutrition (TSB) Only	2.02×10^8	2.1×10^6	1.97×10^8	1.31×10^8
Saline	5.59×10^7	1.02×10^7	5.53×10^7	7.74×10^7
Betadine 1/10	1.24×10^8	5.29×10^5*	8.94×10^6	2.51×10^7
Prontosan 1/10	7.32×10^4*	8.06×10^3**	-	-
Prontosan neat	2.14×10^3**	0***	3.65×10^4*	2.96×10^4*
Rifampicin	9.58×10^6	1.87×10^6	-	-
Gentamicin	-	-	2.06×10^7	1.91×10^6*
